# Generation of tooth–periodontium complex structures using high-odontogenic potential dental epithelium derived from mouse embryonic stem cells

**DOI:** 10.1186/s13287-017-0583-5

**Published:** 2017-06-08

**Authors:** Yancong Zhang, Yongliang Li, Ruirui Shi, Siqi Zhang, Hao Liu, Yunfei Zheng, Yan Li, Jinglei Cai, Duanqing Pei, Shicheng Wei

**Affiliations:** 1 Department of Oral and Maxillofacial Surgery/Central Laboratory, Peking University School and Hospital of Stomatology, National Engineering Laboratory for Digital and Material Technology of Stomatology, Beijing Key Laboratory of Digital Stomatology, Beijing, 100081 People’s Republic of China; 2 Laboratory of Biomaterials and Regenerative Medicine, Academy for Advanced Interdisciplinary Studies, Peking University, Beijing, 100871 People’s Republic of China; 3 Key Laboratory of Regenerative Biology, Guangzhou Institutes of Biomedicine and Health, Chinese Academy of Sciences, Guangzhou, 510530 People’s Republic of China

**Keywords:** Mouse embryonic stem cells, Dental epithelium, BMP4, Tooth regeneration

## Abstract

**Background:**

A number of studies have shown that tooth-like structures can be regenerated using induced pluripotent stem cells and mouse embryonic stem (mES) cells. However, few studies have reported the regeneration of tooth–periodontium complex structures, which are more suitable for clinical tooth transplantation. We established an optimized approach to induce high-odontogenic potential dental epithelium derived from mES cells by temporally controlling bone morphogenic protein 4 (BMP4) function and regenerated tooth–periodontium complex structures in vivo.

**Methods:**

First, immunofluorescence and quantitative reverse transcription-polymerase chain reaction were used to identify the watershed of skin and the oral ectoderm. LDN193189 was then used to inhibit the BMP4 receptor around the watershed, followed by the addition of exogenous BMP4 to promote BMP4 function. The generated dental epithelium was confirmed by western blot analysis and immunofluorescence. The generated epithelium was ultimately combined with embryonic day 14.5 mouse mesenchyme and transplanted into the renal capsules of nude mice. After 4 weeks, the tooth–periodontium complex structure was examined by micro-computed tomography (CT) and hematoxylin and eosin (H&E) staining.

**Results:**

Our study found that the turning point of oral ectoderm differentiation occurred around day 3 after the embryoid body was transferred to a common culture plate. Ameloblastin-positive dental epithelial cells were detected following the temporal regulation of BMP4. Tooth–periodontium complex structures, which included teeth, a periodontal membrane, and alveolar bone, were formed when this epithelium was combined with mouse dental mesenchyme and transplanted into the renal capsules of nude mice. Micro-CT and H&E staining revealed that the generated tooth–periodontium complex structures shared a similar histological structure with normal mouse teeth.

**Conclusions:**

An optimized induction method was established to promote the differentiation of mES cells into dental epithelium by temporally controlling the function of BMP4. A novel tooth–periodontium complex structure was generated using the epithelium.

**Electronic supplementary material:**

The online version of this article (doi:10.1186/s13287-017-0583-5) contains supplementary material, which is available to authorized users.

## Background

Tooth loss (dental disease) is a common condition that greatly affects human health [1, 2]. A common goal of oral medicine is to simulate the mechanism of tooth development to regenerate a whole tooth for clinical tooth replacement [3, 4]. The tooth may become the first commercial organ obtained by regeneration technology due to its visible position and alternative functions [5]. Tooth formation involves a series of complex developmental processes and interactions between epithelial and mesenchymal cells. The regulation of interactions between dental epithelial and mesenchymal cells has mostly been investigated in tooth development research [6–8].

Cells originating from the ectoderm and the cranial neural crest ectomesenchyme interact and convey various signals to induce tooth germ development. Cells differentiate according to their specific functions under the action of signaling molecules in a microenvironment. Although several growth factors and their receptors are expressed in a developing tooth [9–11], bone morphogenic protein 4 (BMP4) is a very important molecule during the initial stage of tooth germ development [12–14]. In early tooth development, BMP4 mediates epithelial–mesenchymal interactions by inducing epithelial signal molecule expression and mesenchymal expression. According to previous studies, the sites of future tooth formation are determined by the antagonistic expression of fibroblast growth factor 8 (FGF8) and BMP4 [15, 16]. A site with high FGF8 expression and low BMP4 expression on embryonic day 10.5 (E10.5) in the mouse oral epithelium tends to develop teeth. The expression of BMP4 concurrently represses that of paired-like homeodomain 2 (PITX2), a marker of the dental epithelium [17, 18]. These data indicate that BMP4 inhibits odontogenesis at the initial stage of tooth generation.

In recent years, a number of studies have focused on tooth regeneration. Otsu et al. [19] established a culture protocol to induce the differentiation of mouse induced pluripotent stem (iPS) cells into neural crest-like cells (NCLCs). iPS cell-derived NCLCs that recombine with the dental epithelium or are cultured in conditioned medium of dental epithelial cells differentiate into dental mesenchymal cells (DMCs) and odontoblasts. Arakaki et al. [20] differentiated dentin sialophosphoprotein (DSPP)-expressing cells by coculturing a mouse dental pulp stem cell line with dental epithelial cells and differentiated ameloblastin (AMBN)-expressing dental epithelial cells by coculturing iPS cells with SF2-24 cells. Fang et al. and Liu et al. [21, 22] successfully generated ameloblast-like cells from mouse iPS cells using ameloblast serum-free conditioned medium (ASF-CM) supplemented with BMP4 to promote odontogenic differentiation. Ochiai et al. [23] found that treatment with BMP4 and FGF8 improved oral ectoderm differentiation from mES cells and that a high BMP4 concentration induces dental epithelium and mesenchyme differentiation. Using custom culture media, Cai et al. [24] differentiated human iPS cells and H1-ES cells into an epithelial sheet that exhibited odontogenic potential when combined with E14.5 mouse dental mesenchyme, although the probability of tooth formation was low. Moreover, Zheng et al. [25] reported that mouse dental mesenchyme lost its odontogenic potential in vitro in the absence of dental epithelial cells. Together, these results suggest that the dental epithelium plays a crucial role during tooth development. Therefore, the generation of a functional dental epithelium should be the basis for tooth regeneration.

Until now, a definite method to induce the differentiation of dental epithelial cells from stem cells has not been reported. To overcome this challenge, we established an optimized strategy based on the nature of tooth development to induce the differentiation of mES cells into dental epithelium by temporally regulating the function of BMP4. The induced dental epithelium exhibited high odontogenic potential and formed tooth–periodontium complex structures that included teeth, a periodontal membrane, and alveolar bone when combined with E14.5 mouse dental mesenchyme.

## Methods

### mES cell culture

mES cells were cultured in KnockOut™ Dulbecco’s Modified Eagle Medium (KO-DMEM; Gibco BRL, Gaithersburg, MD, USA) containing 15% fetal bovine serum (FBS; Gibco BRL), 1% l-glutamine (Gibco BRL), 1% penicillin/streptomycin (Gibco BRL), 1% nonessential amino acids (Gibco BRL), 1% β-mercaptoethanol (Gibco BRL), and 1000 U/ml leukemia inhibitory factor (LIF; Life Technologies, USA) on gelatin precoated plates (Corning, Lowell, MA, USA). The culture medium was changed daily and colonies were evaluated under an inverted microscope (Olympus IX71; Olympus Corp., Tokyo, Japan).

### Differentiation of dental epithelium

A low-attachment plate (Corning) was used to form embryoid bodies (EBs) from mES cells. The mES cells were dissociated into single cells with 0.25% trypsin–ethylene diamine tetraacetic acid (EDTA). Next, 1 × 10^5^ undifferentiated cells/ml were plated and cultured in medium without LIF (MOL): KO-DMEM containing 15% FBS, 1% l-glutamine, 1% penicillin/streptomycin, 1% nonessential amino acids, 2% N_2_ (Invitrogen, Carlsbad, CA, USA), and 1% B27 (Invitrogen). Culture plates were incubated at 37 °C (5% CO_2_, 95% air) for 2 days. Formed EBs were transferred to common culture plates and LDN193189 (Sigma-Aldrich, St. Louis, MO, USA), an antagonist of BMP receptor isotypes ALK2 and ALK3, and BMP4 (Sigma-Aldrich) were added to the medium at the indicated time points (Fig. 2a).

### Quantitative reverse-transcription polymerase chain reaction

Ten aggregates per sample were subjected to quantitative reverse-transcription polymerase chain reaction (qRT-PCR) on an Mx3000P Real-Time PCR System (Agilent Technologies, Santa Clara, CA, USA). Data were normalized to glyceraldehyde-3-phosphate dehydrogenase (GAPDH) expression. The primers used for qRT-PCR are presented in Additional file 1: Table S1.

### Immunofluorescence

Cells grown on culture plates were rinsed with phosphate-buffered saline (PBS), fixed with 4% paraformaldehyde for 10 min at room temperature, and incubated with 10% fetal calf serum (FCS)/PBS for 30 min at 37 °C. Antibodies against the following proteins were used at the indicated dilutions: paired box gene 9 (PAX9; rabbit, 1:200; Santa Cruz Biotechnology, Heidelberg, Germany), PITX2 (mouse, 1:200; Santa Cruz Biotechnology), FGF8 (mouse, 1:200; Santa Cruz Biotechnology), BMP4 (mouse, 1:200; Santa Cruz Biotechnology), and AMBN (mouse, 1:200; Santa Cruz Biotechnology). Cells were incubated with both the primary and secondary antibodies for 30 min at 37 °C and washed with 1× PBS.

### Western blot analysis

The proteins collected from mES cells were separated by 10% sodium dodecyl sulfate-polyacrylamide gel electrophoresis (SDS–PAGE) and subsequently transferred to a polyvinylidene fluoride (PVDF) microporous membrane (Millipore, Boston, MA, USA). After blocking with 5% nonfat milk for 1 h at room temperature, the membrane was incubated for 2 h at room temperature with anti-AMBN (1:500; Santa Cruz Biotechnology), anti-β-catenin (1:500; Santa Cruz Biotechnology), anti-p-Smad1/5/8 (1:500; Santa Cruz Biotechnology), or anti-β-actin (1:1,000; Santa Cruz Biotechnology) antibodies. The blots were subsequently washed with Tween Tris-buffered saline (TTBS; 10 mM Tris–HCl, 50 mM NaCl, and 0.25% Tween) and incubated with the appropriate secondary antibody (Santa Cruz Biotechnology) for 30 min at room temperature.

### In-vivo transplantation assay

Day 14 epithelial cell aggregates were harvested and recombined with E14.5 mouse dental mesenchyme. The combined tissues were transplanted into the renal capsules of nude mice and raised for 4 weeks.

## Results

### Day 3 is the watershed of the oral ectoderm and epidermis

**Fig. 1:**
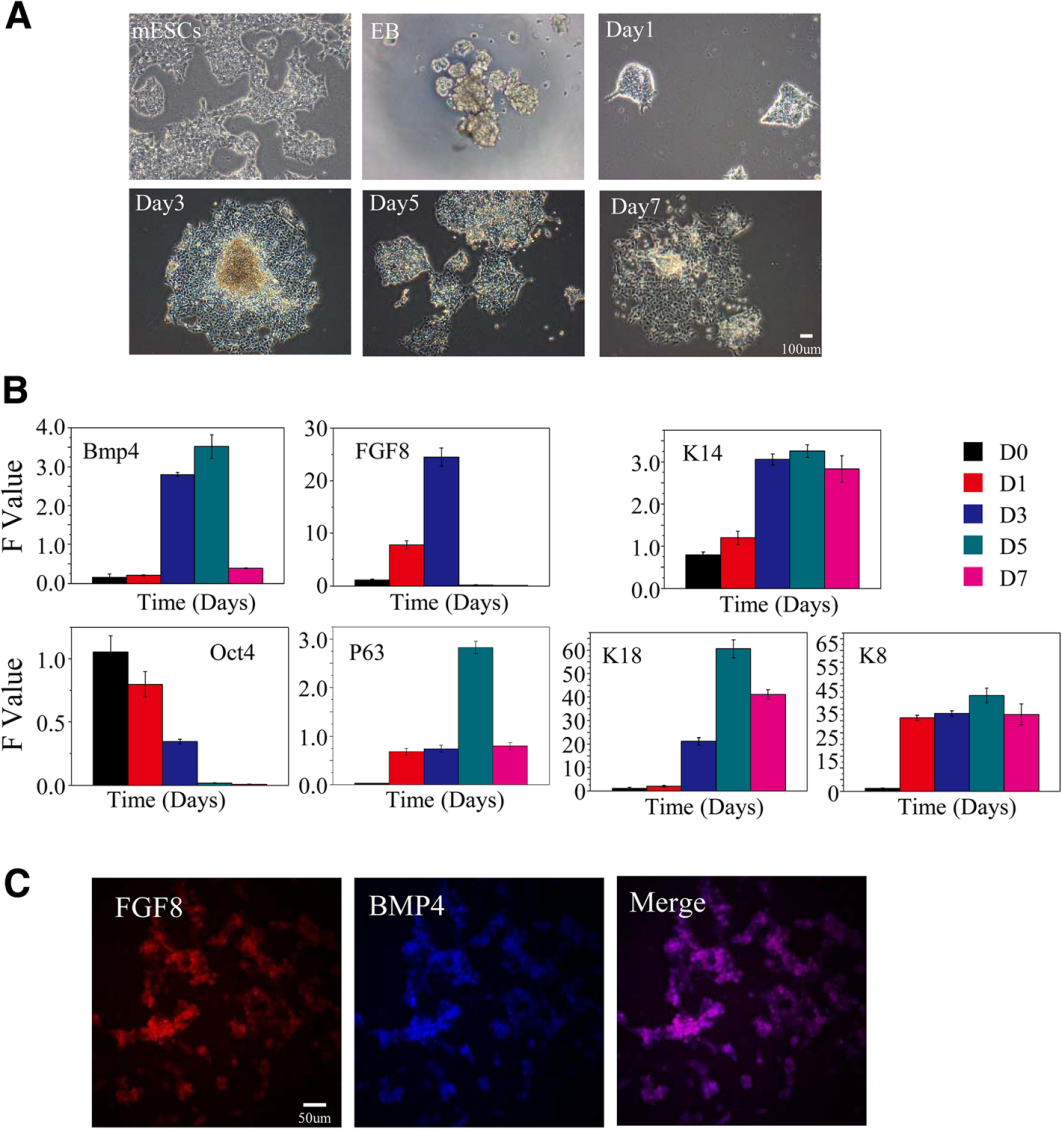
mES cell-derived epithelium. **a** Cell morphology. mES cells present a colony growth character, each clone with an obvious boundary. EBs are round balls. One day after attachment, cells began to climb out peripheral from the EBs (radial growth). A large number of epithelioid cells presented on day 3 and later. **b** qRT-PCR showed representative downregulation of mES cell-specific transcription factor (Oct4) and the expression of keratin epithelial marker k14 was upregulated on day 3. Expression of early ectoderm marker P63 and K18 was relative lower on day 3. FGF8 expression was up regulated on day 3 while BMP4 showed a high expression level. **c** Immunostaining of the day 3 cells for FGF8 and BMP4. *BMP4* bone morphogenic protein 4, *D* day, *EB* embryoid body, *FGF8* fibroblast growth factor 8, *mESC* mouse embryonic stem cell

During early embryonic development, the ectoderm differentiates into the surface ectoderm, neural crest, and neural tube. The surface ectoderm differentiates into skin and the oral ectoderm, and the oral ectoderm gives rise to dental epithelial differentiation [26]. Therefore, the watershed of the oral ectoderm and epidermis that is similar to the E10.5 ectoderm must first be identified in the process of in-vitro cultivation. We first observed cells that moved radially to the periphery of the EBs on day 3 after being transferred to common culture plates. A large number of epithelioid cells were present after 7 days (Fig. 1a). We next tested the expression of a series of epithelial-related genes on days 1, 3, 5, and 7. We did not observe an antagonistic relationship between FGF8 and BMP4. FGF8 expression was upregulated on day 3 whereas BMP4 was highly expressed. In addition, the expression of the keratin epithelial marker K14 was upregulated on day 3. However, expression of the early ectoderm markers p63 and K18 was relatively lower on day 3. These results suggest that mES cells began to differentiate into K14-positive ectoderm, an indication of the epidermal keratinocyte fate, on day 3. This transcription phenotype of these marker genes is similar to that of E10.5, with the exception of BMP4. Therefore, we conclude that the key time point of differentiation to the oral ectoderm or to the epidermis in vitro occurs around day 3 (Fig. 1b). In order to confirm this conclusion, immunofluorescence was taken to test the expression of early oral epithelial markers FGF8 and BMP4 and the expression of early dental epithelial markers PITX2 and early dental mesenchymal marker PAX9. We observed that FGF8 and BMP4 were highly expressed on day 3, the expression of FGF8 and BMP4 overlapped in some areas (Fig. 1c), and PITX2 and PAX9 were not observed (data not shown). These phenomena further confirmed our argument that the key time point of differentiation to the oral ectoderm or to the epidermis in vitro occurs around day 3.

### Generation of AMBN-positive epithelium by temporal manipulation of BMP4 function

**Fig. 2:**
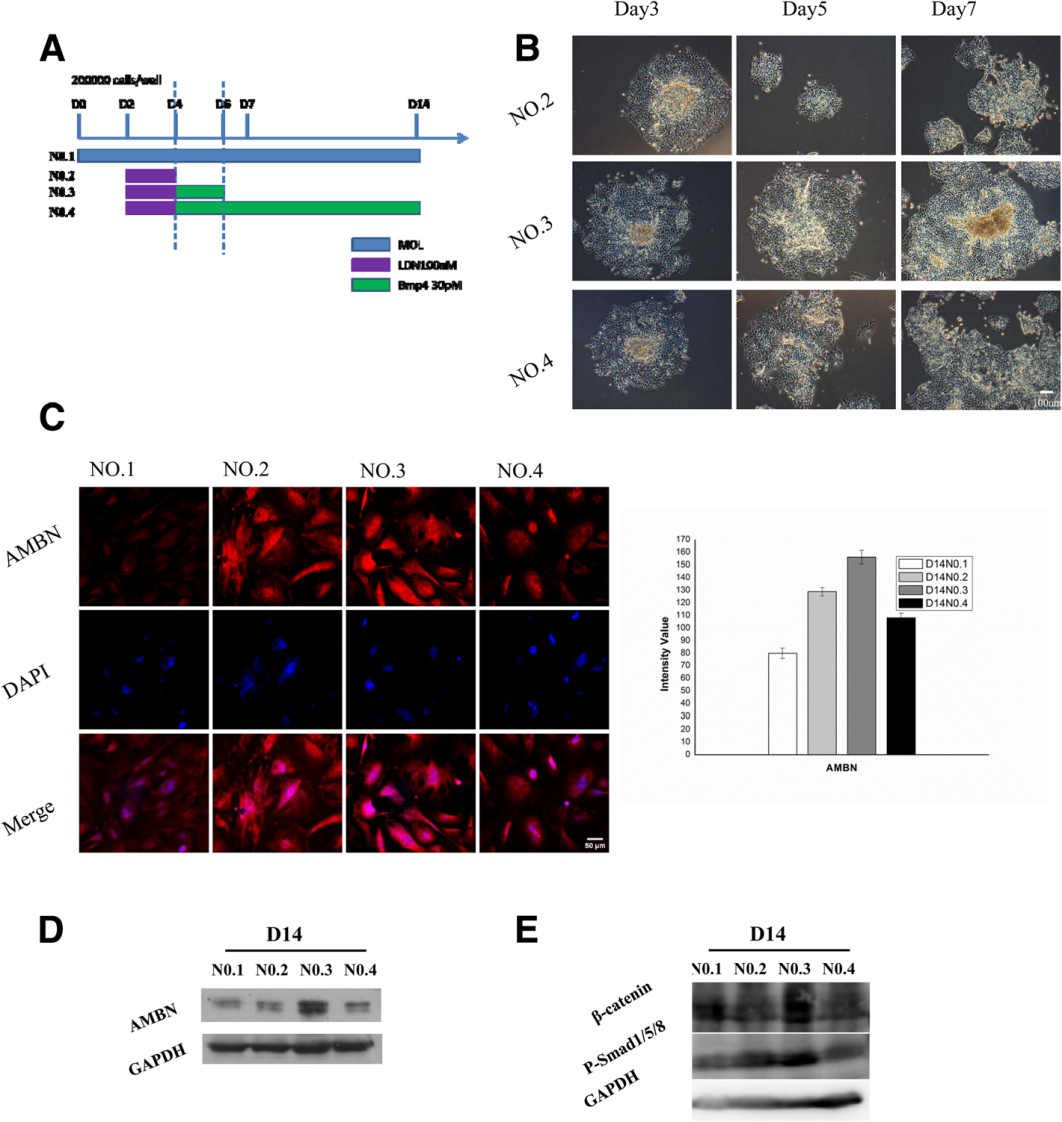
mES cell-derived AMBN-positive cells. **a** mES cell differentiation culture protocols. Protocol No. 1, MOL only; Protocol No. 2, 100 nM LDN193189 added to MOL at the end of day 2 for 2 days; Protocol No. 3, 30 pM BMP4 added to MOL at the end of day 4 for 2 days; and Protocol No. 4, 30 pM BMP4 added to MOL on day 4 for the remaining days. **b** Cell morphologies under different protocols. **c** Immunostaining of the day 14 cells cultured under all protocols. AMBN (*red*), DAPI (*blue*). Thirty-five points were selected randomly from each group for fluorescence intensity analysis (*right*). **d** Western blot for AMBN from mES cells derived epithelial cell. In Protocol No. 3 the AMBN stripe emerged. **e** Western blot images showed significant high expression of P-Smad1/5/8 and β-catenin in Protocol No. 3 as compared with other protocols on day 14 (Color figure online). *AMBN* ameloblastin, *BMP4* bone morphogenic protein 4, *D* day, *GAPDH* glyceraldehyde-3-phosphate dehydrogenase

To mimic the nature of tooth development, we designed and carried out four induction protocols to inhibit the function of BMP4 at the initial stage (days 2–4) following different manipulations of exogenous BMP4 (Fig. 2a): Protocol No. 1, MOL (KO-DMEM containing 15% FBS, 1% l-glutamine, 1% penicillin/streptomycin, 1% nonessential amino acids, 2% N2, and 1% B27) from day 0 to day 14; Protocol No. 2, MOL from day 0 to day 2, then 100 nM LDN193189 (Sigma-Aldrich) was added to MOL at the end of day 2 for 2 days, and at the end of day 4 cells were cultured with MOL for the remaining days; Protocol No. 3, MOL from day 0 to day 2, then 100 nM LDN193189 was added to MOL at the end of day 2 for 2 days, 30 pM BMP4 (Sigma-Aldrich) was added to MOL at the end of day 4 for 2 days, and at the end of day 6 cells were cultured with MOL for the remaining days; and Protocol No. 4, MOL from day 0 to day 2, then 100 nM LDN193189 was added to MOL at the end of day 2 for 2 days, and 30 pM BMP4 was added to MOL at the end of day 4 for the remainder of the experiment. The temporal manipulation of BMP4 (30 pM; days 4–6) resulted in the differentiation of AMBN-positive dental epithelial cells. The expression of AMBN was detected in all groups except that only a small amount of fluorescence was observed in the Protocol No. 1 group. Thirty-five points were selected randomly from each group for fluorescence intensity analysis, and the results showed that AMBN expression in the Protocol No. 3 group was obviously higher than that of other groups (Fig. 2b, c). The results of western blot analysis showed that the amount of AMBN induced by Protocol No. 3 was relatively higher than that induced by the other protocols (Fig. 2d). In the initiation stage of tooth development, multiple Wnt ligands are present in the dental epithelium [27, 28]. This finding suggests that Wnt is likely an early signaling molecule that regulates tooth development. Therefore, we needed to determine whether our method was able to activate the cellular Wnt/Bmp signaling pathway. We selected β-catenin and P-Smad1/5/8 as markers of the Wnt and Bmp pathways, respectively, and examined their expression. Both β-catenin and P-Smad1/5/8 were significantly upregulated using Protocol No. 3 (Fig. 2e).

### Tooth–periodontium complex structures generated from mES cell-derived dental epithelium

**Fig 3:**
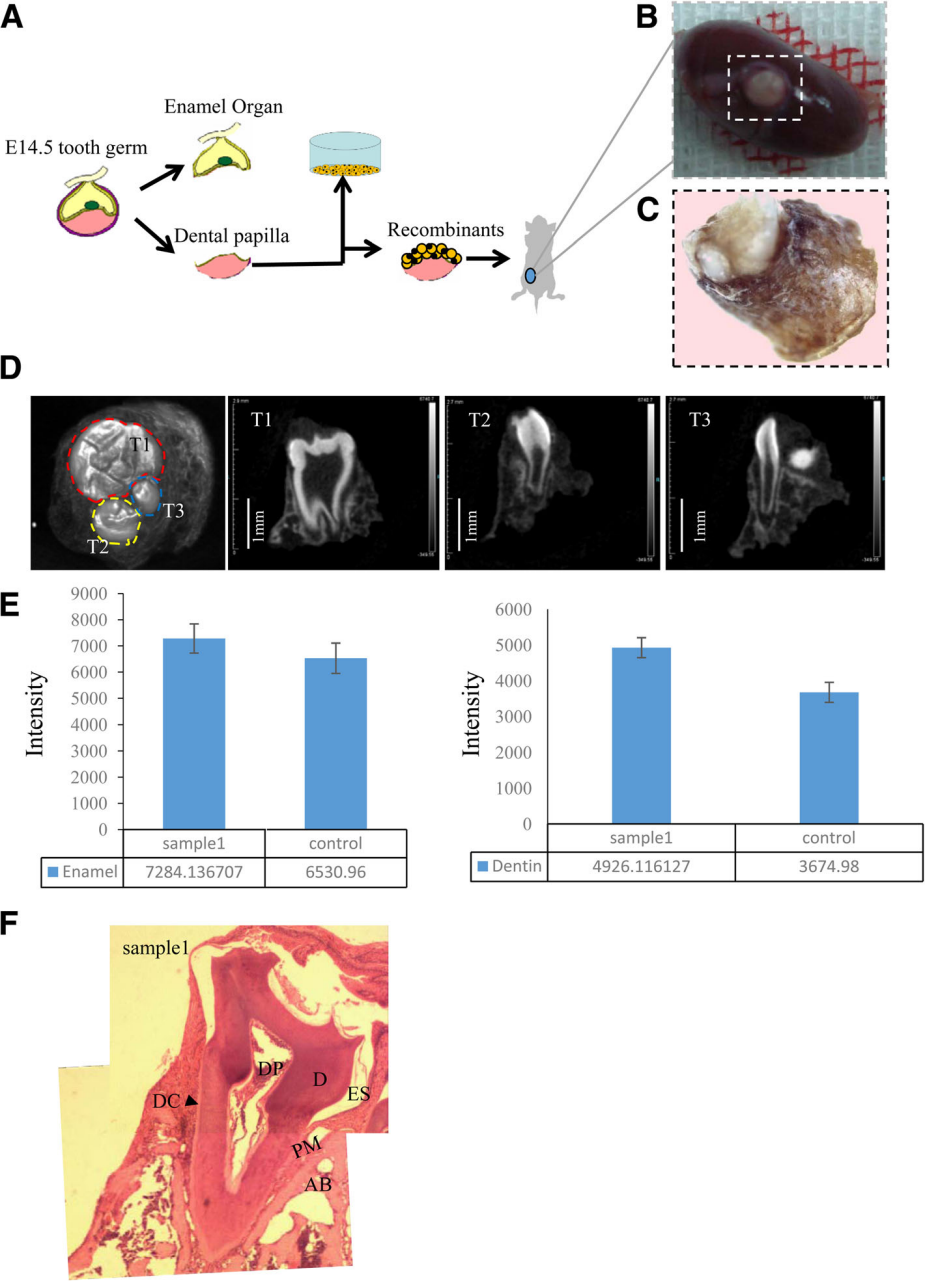
Tooth–periodontium complex structures formed from mES cells. **a** Experimental schematic diagram. **b** Tooth-like structures under the renal capsule. **c** Tooth-like structures under a stereoscopic microscope. **d** Tooth-like structures under micro-CT. Results showed it contained three different teeth types and periodontal tissue. **e** Intensity of the enamel and dentine were higher than normal mouse teeth. **f** HE staining showed tooth-like structures containing dental pulp (*DP*), dentin (*D*), enamel space (*ES*), alveolar bone (*AB*), periodontal membrane (*PM*), and dental cement (*DC*). *E* embryonic day

After obtaining AMBN-positive epithelium, we investigated whether it had high odontogenic potential. Therefore, we combined day 14 epithelium with E14.5 mouse dental mesenchyme, which was then transplanted into the renal capsules of nude mice (Fig. 3a). The surgery was performed on 12 mice; six of the 12 died within 2 days because of surgical trauma. After 4 weeks, tooth-like structures were detected in all six surviving mice (Fig. 3b, c, Additional file 2: Figure S1). The micro-CT analysis revealed three types of teeth and periodontal tissues (Fig. 3d). The intensity of the enamel and dentine was calculated and compared with that of normal mouse teeth (Fig. 3e). The results indicated that the newly generated teeth had a higher level of mineralization relative to normal mouse teeth. Further, H&E staining revealed that the newly generated tooth–periodontium complex contained dentin, dental pulp, enamel space, alveolar bone, a periodontal membrane, and dental cement, similar to normal mouse teeth (Fig. 3f, Additional file 3: Figure S2).

## Discussion

Vertebrate organ development is dependent on inductive interactions between the epithelium and the adjacent mesenchyme [29, 30]. The dental epithelium originates from the oral ectoderm during tooth germ development in vivo. The dental mesenchyme is derived from neural crest cells that form from the ectoderm [31, 32]. Morphological signs of murine tooth development occur on E10–E10.5, when tooth buds exhibit a high level of PAX9 expression (a marker of the site of tooth formation) in the mesenchyme.

A previous study demonstrated that PAX9 was significantly downregulated in the mesenchyme and PITX2 was significantly downregulated in the oral ectoderm in E10.5 mandible when explants were cultured in contact with BMP4-soaked beads; however, they were only slightly affected in late E11.5 and E12.5 [16, 18]. Based on these data, we hypothesized that high expression of BMP4 on E10.5 would repress tooth development (i.e., inhibiting the function of BMP4 on E10.5 would promote tooth formation).

Cells began to move to the periphery of EBs on day 3 of culture in common plates, and after assessing the expression of marker genes of the epidermis and oral ectoderm and tooth development we found that the differentiating cells demonstrated a similar transcription phenotype to that of E10.5 on day 3 of culture in common plates, with the exception of BMP4. We therefore mimicked the principle of tooth development and optimized the culture medium to inhibit the function of BMP4 at the end of day 2 for 48 h. Following incubation for 48 h, exogenous BMP4 was added to promote cell proliferation and generated AMBN-positive epithelium on day 14. The effect of time on exogenous BMP4 treatment was evaluated by examining the expression of AMBN. Treatment with BMP4 for 48 h led to relatively high expression of AMBN on day 14 compared with the other protocols. Further examination of the expression of two marker proteins of the Wnt/Bmp signaling pathway indicated that treatment with exogenous BMP4 for 48 h activated the cellular Wnt/Bmp pathway. AMBN is a marker of ameloblasts [33]. Although previous studies have reported that AMBN-positive epithelium differentiates from iPS cells and mES cells in conditioned medium derived from ameloblasts, a definite method for the differentiation of AMBN-positive epithelium has not been reported. Therefore, our study provides new insight into the differentiation of AMBN-positive epithelium in vitro.

Finally, to test the odontogenic potential of the epithelium, we combined day 14 AMBN-positive epithelium with E14.5 mouse dental mesenchyme and found that the epithelium exhibited high odontogenic potential, with all living mice possessing tooth-like structures. Micro-CT analysis indicated not only that the tooth structure formed but that a periodontal structure was also generated. H&E staining revealed that the tooth–periodontium complex contained dentin, dental pulp, enamel space, alveolar bone, a periodontium membrane, and dental cement. To our knowledge, this type of tooth–periodontium complex structure is the first to be reported. Therefore, we believe that this complex structure will be more suitable for tooth regeneration and transplant because the healing of bone to bone is easier than that of tooth root to bone.

Future work should focus on human pluripotent stem cells, which could ultimately be applied to the clinical regeneration of human teeth; however, whether this research also applies to human pluripotent stem cells remains to be resolved.

## Conclusions

In summary, we established an optimized strategy to induce the differentiation of dental epithelium from mES cells in vitro. AMBN-positive dental epithelium was differentiated by treatment with exogenous BMP4 for an appropriate time after initial inhibition. Combined with E14.5 mesenchyme, the epithelium exhibited high odontogenic potential and generated tooth–periodontium complex structures.

## Additional files

Additional file 1: Table S1. presenting the primers used for qPCR. (DOCX 13 kb)

Additional file 2: Figure S1. showing tooth-like structures under the renal capsule. Tooth-like structures were detected from all six surviving mice. (PNG 2401 kb)

Additional file 3: Figure S2. showing normal mouse tooth structures. H&E staining (*right*) revealed that the normal mouse tooth structures contained dental pulp (*DP*), dentin (*D*), enamel space (*ES*), alveolar bone (*AB*), periodontal membrane (*PM*), and dental cement (*DC*). Normal mouse molar structures and periodontal tissues under micro-CT (*left*). (PNG 3132 kb)

## Abbreviations

ASF-CM: ameloblast serum-free conditioned medium
DMC: Dental mesenchymal cell
DSPP: Dentin sialophosphoprotein
EB: Embryoid body
H&E: hematoxylin and eosin
iPS: Induced pluripotent stem
mES: mouse embryonic stem
NCLC: Neural crest-like cell
qRT-PCR: quantitative reverse transcription-polymerase chain reaction
