# Supplementary material for: Generation of tooth–periodontium complex structures using high-odontogenic potential dental epithelium derived from mouse embryonic stem cells
Source: Stem Cell Res Ther. 2017 Jun 8;8:141. doi: 10.1186/s13287-017-0583-5 (PMC5465544; doi:10.1186/s13287-017-0583-5)
Supplement: Supplementary file 1 — presenting the primers used for qPCR. (DOCX 13 kb) [file 13287_2017_583_MOESM1_ESM.docx]

TableS1 Primers used for qPCR

| Gene | Primer sequence |
| --- | --- |
| K18 | Foward:TTGTCACCACCAAGTCTGCC |
|  | Reverse:TTTGTGCCAGCTCTGACTCC |
| K8 | Forward:GTGTCCACTGGCGATGTGAACGTGG |
|  | Reverse:GCTGCCGCAGTAGCGACTCTACTGT |
| K14 | Forward:GTGAAGATTCGGGACTGGTAC |
|  | Reverse:TCTGCTCCGTCTCAAACTTG |
| P63 | Forward:ACGCCCCGCCTCTTTGCAAAT |
|  | Reverse:TGAGCTGGGGTTTCTATGAAACGCT |
| FGF8 | Forward:GACAGGTCTCTACATCTGCATG |
|  | Reverse:TTGTTCTCCAGCACGATCTC |
| Bmp4 | Forward:GATGGGATTCTCGTCTAAACCG |
|  | Reverse:CAAACTTGCTGGAAAGGCTC |
| Oct4 | Forward:CACTCTACTCAGTCCCTTTTCC |
|  | Reverse:GTTCTCTTGTCTACCTCCCTTG |
| β-Actin | Forward:GTTTGAGACCTTCAACACCCC |
|  | Reverse:GTGGCCATCTCCTGCTCGAAGTC |
