# Supplementary figures and images for: Generation of tooth–periodontium complex structures using high-odontogenic potential dental epithelium derived from mouse embryonic stem cells
Source: Stem Cell Res Ther. 2017 Jun 8;8:141. doi: 10.1186/s13287-017-0583-5 (PMC5465544; doi:10.1186/s13287-017-0583-5)

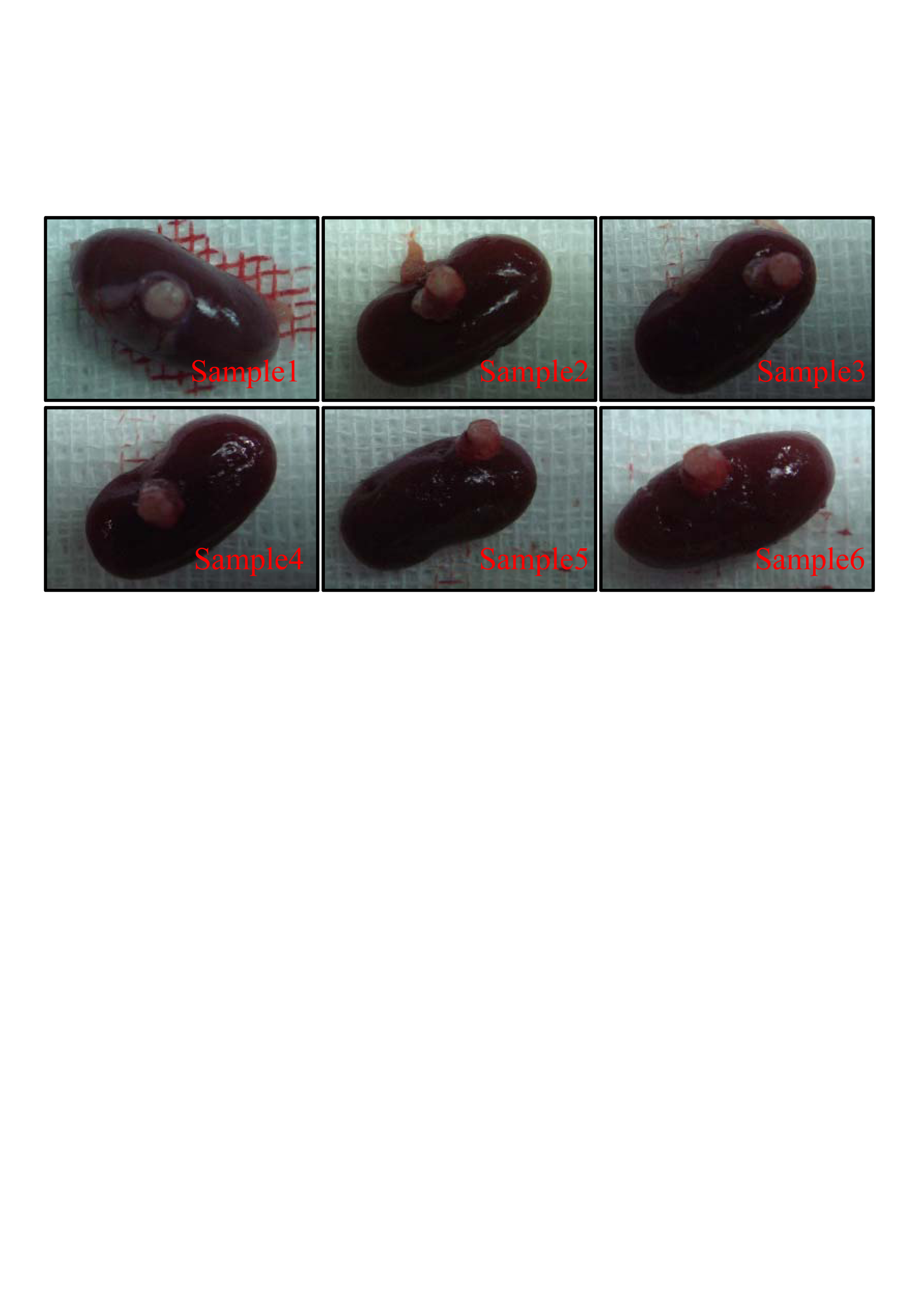

Supplement: Supplementary file 2 — showing tooth-like structures under the renal capsule. Tooth-like structures were detected from all six surviving mice. (PNG 2401 kb) [file 13287_2017_583_MOESM2_ESM.png]

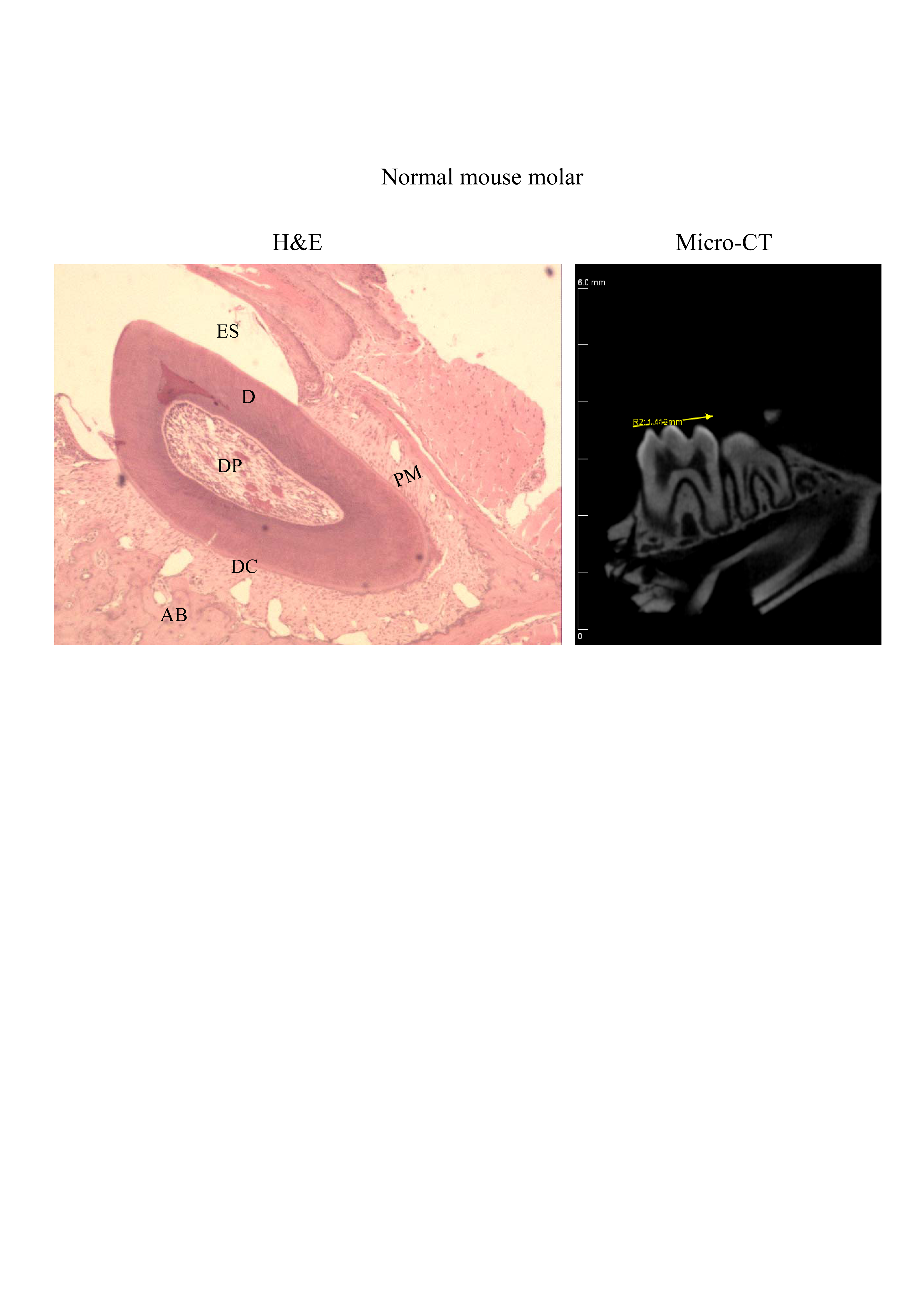

Supplement: Supplementary file 3 — showing normal mouse tooth structures. H&E staining (right) revealed that the normal mouse tooth structures contained dental pulp (DP), dentin (D), enamel space (ES), alveolar bone (AB), periodontal membrane (PM), and dental cement (DC). Normal mouse molar structures and periodontal tissues under micro-CT (left). (PNG 3132 kb) [file 13287_2017_583_MOESM3_ESM.png]
